# Supplementary material for: Content-rich biological network constructed by mining PubMed abstracts
Source: BMC Bioinformatics. 2004 Oct 8;5:147. doi: 10.1186/1471-2105-5-147 (PMC528731; doi:10.1186/1471-2105-5-147)
Supplement: Additional File 5 — The original Chilibot query results of the term "long-term potentiation (LTP)" and 22 other terms, limiting the latest references analyzed to the years 1990, 1995, 2000, and 2004. [file 1471-2105-5-147-S5.bz2 › chilibotAdditionalFile5/ltp2000/html/CREB.html]

 


**CREB** (Input: CREB ) 

---


**Google Searches:** Entire Web  | EDU domain only  | PDF files only

.

**External Links:** OMIM |
LocusLink
| Swissprot
| GeneCards

  
**Maps of CREB**

Simple
Complete
graph in
radiant
tree
square
layout.

**New Hypothesis !**

**Synonyms** 

- creb   [PubMed]

**Synopsis**

- **CREB** is a hormonally stimulated transcriptional activator, and CaM kinase may lie on the pathway to its activation.  Annu Rev Biochem, 1992 
  [28]

- Both pCREB immunocytochemical staining and Western blotting showed that **CREB** is rapidly and transiently phosphorylated after induction of long term potentiation  [LTP] .  J Neurosci Methods, 1997 
  [26]

- We, and others, have shown that this transcriptional switch is conserved, at least in part, in the regulation of synaptic plasticity in mice **CREB** is implicated in activation of genes required for LTP, a model for synaptic plasticity in the mammalian hippocampus.  C R Acad Sci IIIC R Acad Sci III, 1997 
  [25]

- Together, we have resolved the spatial and temporal dynamics of **CREB** phosphorylation during hippocampal LTP, showing that the transcription factor **CREB** is specifically recruited at two distinct time points in some forms of hippocampal synaptic plasticity in vivo.  J Neurosci, 1999 
  [23]

- CaMKIV dependent activation of **CREB** is a key event in the expression of genes involved in the processes of T cell activation and neuronal long term potentiation  [LTP] .  Biometals, 1998 
  [22]

- These studies suggests that **CREB** plays a role in experience dependent plasticity in the adult neocortex.  Cereb CortexCereb Cortex, 1998 
  [21]

- These results suggest that NO contributes to L LTP by stimulating guanylyl cyclase and cGMP dependent protein kinase, which acts in parallel with PKA to increase phosphorylation of the transcription factor **CREB**.  J Neurosci, 1999 
  [20]

- Our studies indicate a critical role for the MAPK cascade in the regulation of **CREB** phosphorylation in the hippocampus.  Acta Neurobiol Exp (Wars), 2000 
  [19]

- These findings indicate that upregulation of **CREB** is a common action of chronic antidepressant treatments that may lead to regulation of specific target genes, such as BDNF and trkB, and to the long term effects of these treatments on brain function.  J Neurosci, 1996 
  [19]

- Genetic approaches have established that the transcription factor **CREB** is essential for long term memory.  Curr Opin Neurobiol, 1995 
  [17]

- Thus, MAPK ERK activation is required for LTP dependent transcriptional regulation and we suggest this is regulated by two parallel signaling pathways, the MAPK ERK Elk 1 pathway targeting SRE and the MAPK ERK **CREB** pathway targeting CRE.  J Neurosci, 2000 
  [16]

- Immunocytochemical studies show that both repeated tetanization and application of forskolin stimulate the phosphorylation of cAMP response element binding proteins **CREB** in cells of the lateral nucleus of the amygdala.  J Neurosci, 2000 
  [16]

- Experiments with a series of simplified reporter genes in combination with specific recombinant protein kinase inhibitors suggest that induction of gene activity following NO amplified calcium action involves protein kinase A dependent activation of the transcription factor **CREB**.  Nature, 1993 
  [16]

- Furthermore, the full expression of the late phase of long term potentiation  [LTP]  L LTP and L LTP associated CRE mediated transcription requires ERK activation, suggesting that the activation of **CREB** by ERK plays a critical role in the formation of long lasting neuronal plasticity.  Neuron, 1998 
  [16]

- the persistence of clear behavioral deficits as shown by the F1 hybrid CREBcomp mice in water maze and fear conditioning indicates a robust and repeatable phenomenon that will permit further functional analysis of **CREB**.  Learn MemLearn Mem, 1998 
  [16]
